# Ernährungsberatung in ärztlichen Praxen verschiedener Fachrichtungen – eine Querschnittsstudie

**DOI:** 10.1007/s00103-024-03870-0

**Published:** 2024-04-19

**Authors:** Hannah Luisa Mertens, Andrea Kaifie

**Affiliations:** Institut für Arbeits‑, Sozial- und Umweltmedizin, Uniklinik RWTH Aachen, Pauwelsstraße 30, 52074 Aachen, Deutschland

**Keywords:** Niedergelassene Ärzte, Wissen, Einstellung, Verhalten (Gesundheit), Präventivmedizin, Nichtübertragbare Krankheiten, Erhebungen und Fragebögen, Primary Care Physicians, Health Knowledge, Attitudes, Practice, Preventive Medicine, Noncommunicable Diseases, Surveys and Questionnaires

## Abstract

**Hintergrund:**

Ziel dieser Querschnittsstudie war es, den Stand der Ernährungsberatung (EB) und Versorgung in ärztlichen Praxen zu erheben. Das entsprechende Fachwissen der Ärzt:innen sowie Hürden und Verbesserungsmöglichkeiten für die EB wurden analysiert.

**Methoden:**

Ein Fragebogen mit 32 Items wurde erstellt und nach 2‑stufigem Pretest im Frühjahr 2022 an alle niedergelassenen Ärzt:innen der Fachrichtungen Allgemeinmedizin, Innere Medizin, Gynäkologie, Orthopädie und Arbeitsmedizin in Aachen (*N* = 360) verteilt.

**Ergebnisse:**

Die Rücklaufquote lag bei 29,2 % (*n* = 105). 85,7 % der Ärzt:innen führten während ihrer Sprechstunde EB durch. Die Ärzt:innen schätzten, dass 39,5 % ihrer Patient:innen EB benötigten, tatsächlich beraten wurden aber nur 28,1 %. Bei Allgemeinmediziner:innen dauert die EB durchschnittlich 13 min, bei allen anderen 9,5 min. Die meisten Teilnehmer:innen fühlten sich gut über gesunde Ernährung informiert (95,2 %) und schrieben dem Thema eine hohe persönliche (57,7 %) und berufliche (47,6 %) Relevanz zu.

Die größten Hürden für eine bessere EB waren die mangelnde Vermittlung von Wissen und Kompetenzen im Medizinstudium (89,5 %) und in der Facharztweiterbildung (79,6 %) sowie Zeitmangel (75,2 %). Viele Teilnehmer:innen würden mehr EB durchführen, wenn diese Leistung besser bezahlt würde und im Arbeitsalltag mehr Zeit zur Verfügung stände.

**Diskussion:**

Die hohe Relevanz von EB ist vielen niedergelassenen Ärzt:innen bewusst, der Bedarf an EB wird von ihnen jedoch nicht gedeckt. Um EB in ärztlichen Praxen zu fördern, bedarf es unter anderem einer besseren ernährungsmedizinischen Ausbildung, einer angemessenen Vergütung und ausreichender Zeitkontingente.

## Abstract (en)

**Introduction:**

The purpose of this study was to assess the status of nutrition care and counseling in German medical practices, including physicians’ nutrition interest and knowledge, existing obstacles, and possible ways of improving nutrition care.

**Methods:**

After development and a two-stage pretest, questionnaires with 32 items were distributed to all resident doctors of general medicine, internal medicine, gynecology and obstetrics, orthopedics, and occupational medicine in Aachen (*n* = 360) in early spring 2022.

**Results:**

The response rate was 29.2% and *n* = 105 participants were included in the analysis. During their consultation hours, 85.7% of the doctors conducted nutrition counseling (NC). While doctors estimated that 39.5% of their patients need NC, they counseled only 28.1%. General practitioners spent an average of 13 min on NC; all other specialties spent 9.5 min. Most participants felt well informed about healthy nutrition (95.2%). Nutrition was of high personal interest to 57.7% of the participants, and 47.6% considered it important for their work as a doctor. Barriers preventing better NC included the lack of nutrition education during medical studies (89.5%) and specialist training (79.6%), and the lack of time (75.2%). Most participants were willing to conduct more NC if they were paid better and had more time for NC during their working hours.

**Discussion:**

Doctors consider NC a relevant part of their work, despite many barriers. To make better use of NC as a measure for treating and preventing common diseases, improvements like better nutrition education for doctors, better funding, and more time for NC are required.

## Einleitung

Ungesunde Ernährung und Bewegungsmangel sind die Hauptursachen für Übergewicht, Adipositas und die damit einhergehenden Gesundheitsrisiken. Ein hoher Body-Mass-Index (BMI) geht einher mit einem erhöhten Risiko für die häufigsten nichtübertragbaren Krankheiten, wie Diabetes, Herz-Kreislauf-Erkrankungen, verschiedene Krebsarten wie Darm‑, Speiseröhren‑, Gallenblasen‑, Gebärmutter- und Brustkrebs, Arthrose und psychische Erkrankungen wie Depressionen oder Demenz [1]. Die Sterblichkeitsrate von Menschen, die an Adipositas oder Übergewicht leiden, ist im Vergleich zu Normalgewichtigen deutlich erhöht [2]. Jeder 5. weltweite Todesfall lässt sich auf ernährungsbedingte Risikofaktoren zurückführen. Eine ungesunde Ernährung ist somit einer der wichtigsten Risikofaktoren für vorzeitiges Versterben [3].

Die Prävalenz von Übergewicht hat sich in den vergangenen 50 Jahren fast verdreifacht: 39 % der Weltbevölkerung [4] und mehr als zwei Drittel der deutschen Bevölkerung [5] sind übergewichtig. Infolgedessen steigen die mit Übergewicht verbundenen Ausgaben des Gesundheitswesens stark an und die Frage, wie diese große Belastung für die sozialen Gesundheitssysteme verringert werden kann, gewinnt zunehmend an Bedeutung [6].

Neben reiner Verhaltensprävention mit Fokus auf Bewegung und Ernährung [7] sind deshalb auch verhältnispräventive Maßnahmen zu berücksichtigen. Viel Potenzial bieten hier politische und gesellschaftliche Initiativen zur Bewegungsförderung und zur Erleichterung einer gesunden Ernährung, beispielsweise eine entsprechende Besteuerung ungesunder Lebensmittel oder die verpflichtende Umsetzung von Qualitätsstandards in Kantinen und Mensen [8, 9]. Derartige Maßnahmen können sich auf den Gesundheitszustand der Bevölkerung positiv auswirken und das Bewusstsein für eine gesündere Ernährung langfristig fördern.

Ein wichtiges Element zur Förderung von gesunder Ernährung im Rahmen der Verhaltensprävention ist Ernährungsberatung. „Ernährungsberatung“ ist in Deutschland kein gesetzlich geschützter Begriff. In Fachkreisen wird unterschieden zwischen „Ernährungsberatung“ als primärpräventiver Leistung und „Ernährungstherapie“ zur Sekundär- und Tertiärprävention sowie zur Behandlung von ernährungsassoziierten Krankheiten. Beide Leistungen sollten nur von speziell qualifizierten Ernährungsfachkräften wie Diätassistent:innen, Oecotropholog:innen und Ärzt:innen mit Zusatzbezeichnung Ernährungsmedizin durchgeführt werden [10, 11]. Der initiale Anknüpfungspunkt für Patient:innen mit ernährungsassoziierten Gesundheitsrisiken und -problemen sind jedoch meistens niedergelassene Ärzt:innen [12], die häufig keine spezielle ernährungsmedizinische Qualifikation haben. Der Umgang dieser Ärzt:innen mit dem Thema Ernährung ist oftmals entscheidend. Wenn sie sich der hohen Relevanz von Ernährung für die Gesundheit der Patient:innen bewusst sind, können sie die Patient:innen gezielt auf ihre Ernährung ansprechen, sie zu Veränderungen motivieren und bei Bedarf zur professionellen Ernährungsberatung oder -therapie an Ernährungsfachkräfte weiterleiten [11].

Ernährungsberatungen in ärztlichen Praxen – im Sinne des Patient:innengesprächs über Ernährung ambulant tätiger Ärzt:innen ohne spezielle ernährungsmedizinische Qualifikationen – sind Gegenstand aktueller internationaler Forschung, insbesondere in den USA und Australien. International wird dieses Format der Ernährungsberatung von den meisten Ärzt:innen als sehr wichtig erachtet [13, 14], sie wird jedoch nur bei einer kleinen Anzahl von Patient:innen durchgeführt und dauert im Durchschnitt weniger als 3–5 min [15–18]. Darüber hinaus werden die Ausbildung zu Ernährung und die Vermittlung von Kompetenzen in der Beratung in der ärztlichen Ausbildung oft als unzureichend eingeschätzt [13, 19, 20]. Zeitmangel, mangelnde Compliance der Patient:innen und unzureichende finanzielle Vergütung stellen weitere Probleme dar [14, 21].

Die meisten internationalen Studien zur Ernährungsberatung in ärztlichen Praxen sind aufgrund der unterschiedlichen Gesundheitssysteme nur eingeschränkt auf Deutschland übertragbar. Die Studien beziehen sich vordergründig auf die Allgemeinmedizin [13–15, 18, 21], einige auch auf Innere Medizin [16, 20, 22]. Abgesehen von Kushner, der Kinderärzt:innen befragte [17], und Smith et al, die die Gynäkologie und Geburtshilfe [19] einbeziehen, existieren kaum Informationen über Ernährungsberatung durch Ärzt:innen anderer Fachrichtungen. Die Folgen ungesunder Ernährung betreffen jedoch viele medizinische Fachrichtungen. So stehen Arthrose als orthopädisches Krankheitsbild sowie gynäkologische Krebsarten in engem Zusammenhang mit Adipositas [4].

Die letzte deutsche Studie zu Ernährungsberatung in ärztlichen Praxen liegt mit Ausnahme einer Studie bei Krebstherapie [23] bereits mehr als 20 Jahre zurück [22]. Es ist somit nur unzureichend erforscht, wie niedergelassene Ärzt:innen zu Beratungen über Ernährung stehen und inwieweit sie diese durchführen, zumal es in den letzten Jahren viele Veränderungen im deutschen Gesundheitssystem gegeben hat, beispielsweise die Umsetzung eines neuen Gesetzes zur Gesundheitsförderung und Prävention im Jahr 2015 [24].

Ziel dieser Studie ist es, den aktuellen Stand zur Ernährungsberatung nicht ausschließlich speziell in Ernährungsmedizin geschulter Ärzt:innen in ärztlichen Praxen unterschiedlicher Fachrichtungen zu erheben. Dies beinhaltet Dauer, Häufigkeit und Inhalte der Ernährungsberatung sowie das Wissen der Ärzt:innen und ihr Interesse an ernährungsmedizinischen Themen. Darüber hinaus werden Probleme und Verbesserungsmöglichkeiten der ernährungsbezogenen Versorgung erfasst und Unterschiede zwischen den Facharztrichtungen analysiert.

## Methoden

Die vorliegende Querschnittsstudie wurde in der Stadt Aachen (Einwohnerzahl ~250.000) durchgeführt. Die Daten wurden mit einem anonymisierten Fragebogen in Papierform erhoben. Alle niedergelassenen Ärzt:innen der Fachrichtungen Allgemeinmedizin, Innere Medizin, Gynäkologie und Geburtshilfe, Orthopädie und Arbeitsmedizin in Aachen wurden über die Website der Kassenärztlichen Vereinigung ermittelt und zur Teilnahme aufgerufen (*N* = 360). Die Fragebögen wurden im Januar und Februar 2022 in den jeweiligen Arztpraxen persönlich abgegeben, nachdem einige Tage zuvor eine E‑Mail zur Information an alle Praxen mit verfügbaren E‑Mail-Adressen versendet wurde. Die ausgefüllten Fragebögen wurden in vorfrankierten Umschlägen per Post zurückgesendet, um Anonymität zu gewährleisten. Es wurde kein Anreiz für die Teilnahme gesetzt.

Vor Beginn der Befragung wurde ein 2‑stufiger Pretest des Fragebogens mit 15 Teilnehmer:innen durchgeführt und es wurden kleinere Anpassungen vorgenommen. Die Ethikkommission der Rheinisch-Westfälischen Technischen Hochschule Aachen hat zu der Studie eine positive Stellungnahme abgegeben (EK 441-21).

### Aufbau des Fragebogens

Die Datenerhebung erfolgte mittels eines aus 32 Fragen bestehenden Fragebogens, der Fragen und Stellungnahmen zu den folgenden Themen enthielt:

1. demografische Daten und allgemeine Informationen: Geschlecht, Alter, medizinisches Fachgebiet, Art der Praxis, durchschnittliche Arbeitszeit, Fortbildungen zum Thema Ernährung,
2. Einstellung zu gesunder Ernährung,
3. Informationsquellen über Ernährung,
4. selbst eingeschätztes Wissen über gesunde Ernährung und verschiedene Ernährungsformen,
5. Haltung zu Ernährungsberatung und daraus entstehenden Aufgaben für Ärzt:innen,
6. Ernährungsberatung in ärztlichen Praxen: Durchführung von professioneller Ernährungsberatung, Notwendigkeit, Häufigkeit, Dauer, Anlass und Inhalt der Ernährungsberatung,
7. mögliche Probleme der Ernährungsberatung: Wirksamkeit, Ausbildung und Motivation der Ärzt:innen, verfügbare Zeit, Vergütung,
8. erforderliche Veränderungen für eine bessere Ernährungsberatung.

Um Missverständnisse zu vermeiden, definierten wir auf dem Fragebogen vorab das allgemeine Patient:innengespräch über Ernährung im Rahmen der alltäglichen Sprechstunde als „Ernährungsberatung“. Die in der Rahmenvereinbarung zur Qualitätssicherung in der Ernährungsberatung/-therapie und Ernährungsbildung in Deutschland [10] definierten Begriffe Ernährungsberatung und Ernährungstherapie als umfassende Leistungen, die nur von speziell qualifiziertem Fachpersonal durchgeführt werden dürfen, fassten wir im Fragebogen zur besseren allgemeinen Verständlichkeit als „professionelle Ernährungsberatung“ zusammen. Entsprechend verwenden wir die Begriffe auch im Manuskript wie im Fragebogen definiert.

Zu den Fragetypen des Bogens gehörten binäre Fragen, Multiple-Choice-Fragen, offene Fragen und 4‑stufige Likert-Skalen. 4‑stufige Likert-Skalen wurden gewählt, um neutrale Antworten zu vermeiden und Tendenzen sichtbar zu machen.

### Statistische Auswertung

Kontinuierliche Daten wurden als Mittelwert ± SD oder Median (Q1–Q3) bei stark verzerrten Daten angegeben. Kategoriale Ergebnisse wurden als absolute und relative Häufigkeiten (%) angegeben. Zusammenhänge zwischen kontinuierlichen Variablen wurden mit dem Pearson-Korrelationskoeffizienten, Assoziationen zwischen Likert-Skalen und ordinalen oder kontinuierlichen Variablen mit dem Spearman-Korrelationskoeffizienten analysiert. Kontinuierliche Variablen in Bezug auf binäre Merkmale wurden mit T‑Tests ausgewertet, Likert-Skalen in Abhängigkeit von binären Variablen mit Mann-Whitney-U-Tests und Likert-Skalen in Abhängigkeit von nominalen, nichtbinären Variablen mit Kruskal-Wallis-Tests. Die erforderlichen Annahmen wurden in allen Fällen geprüft und erfüllt. Für die fachgruppenspezifischen Analysen wurden die Facharztgruppen Orthopädie und Arbeitsmedizin zu Sonstige zusammengefasst. Im Falle von Mehrfachvergleichen wurden die *p*-Werte angepasst. Das Signifikanzniveau wurde auf 0,05 festgelegt.

Die statistischen Analysen wurden mit der Statistiksoftware SAS Enterprise Edition 3.81 (SAS Institute Inc., Cary NC, USA, 2022) durchgeführt.

## Ergebnisse

Insgesamt 105 Ärzt:innen (Rücklaufquote = 29,2 %) nahmen an der Studie teil. 58,1 % der Teilnehmenden waren weiblich, 41,9 % waren männlich. Das Durchschnittsalter betrug 52,3 ± 10,8 Jahre. Die größte Gruppe der Befragten waren Allgemeinmediziner:innen (41,0 %), gefolgt von Fachärzt:innen für Innere Medizin (26,7 %) und Gynäkologie (22,9 %). Von allen befragten Facharztgruppen hatte die Gynäkologie die höchste Antwortrate (36,4 %), während nur 22,2 % aller Orthopäd:innen die Fragen beantworteten (Tab. 1).

**Tab. 1:** Antwortrate in Abhängigkeit von soziodemografischen Merkmalen und der medizinischen Fachrichtung der Teilnehmenden (*n* = 105)

	Teilnehmende	Antwortrate (%)
*Teilnehmende insgesamt*	105	29,2
*Alter (Jahre; Mittelwert (SD))*	52,3 (10,8)	–
*Geschlecht (n; %)*		
Männlich	44 (41,9)	26,7
Weiblich	61 (58,1)	31,3
Divers	0 (0)	–
*Medizinische Fachrichtung (n; %)*		
Allgemeinmedizin	43 (41,0)	28,3
Innere Medizin	28 (26,7)	27,5
Gynäkologie und Geburtshilfe	24 (22,9)	36,4
Orthopädie	6 (5,7)	22,2
Arbeitsmedizin	4 (3,8)	30,8

9,5 % der Teilnehmenden führten die Zusatzbezeichnung Ernährungsmedizin und 11,4 % haben eine Weiterbildung zur Ernährungsmedizinischen Grundversorgung absolviert. Die Mehrheit der Befragten (57,1 %) arbeitete in einer Gemeinschaftspraxis mit im Median 3 Ärzt:innen, 42,9 % arbeiteten in einer Einzelpraxis. Die Arbeitszeit betrug im Durchschnitt 39,7 ± 11,0 h pro Woche (Tab. 2). Wie im Vorfeld geplant, wurden die Gruppen Orthopädie und Arbeitsmedizin für die statistische Auswertung zu „Sonstige“ zusammengefasst.

**Tab. 2:** Angaben der Ärzt:innen zu ihrem Arbeitsplatz, Fortbildungen und Relevanz des Themas Ernährung

*Arbeitszeit/Woche (Mittelwert (SD))*	39,7 (11,0)
*Arbeitsplatz (%)*	
Einzelpraxis	42,9
Gemeinschaftspraxis	57,1
*Anzahl Ärzt:innen in der Gemeinschaftspraxis (Median (Q1; Q3))*	3 (2; 4)
*Teilnehmer:innen mit Fortbildung in ernährungsmedizinischer Grundversorgung (%)*	11,5
*Teilnehmer:innen mit Zusatzbezeichnung Ernährungsmedizin (%)*	9,6
*Anteil der für Ernährungsthemen aufgewendeten Fortbildungszeit (%)*	
Keine	5,8
Weniger als 10 %	59,2
10–30 %	29,1
Mehr als 30 %	5,8
*Wichtigste Informationsquellen zum Thema Ernährung (%)*	
Keine	1,0
Deutsche Gesellschaft für Ernährung	25,7
Offizielle Leitlinien	41,9
Fortbildungen/Tagungen/Kongresse	55,2
Fachzeitschriften	70,5
Fachwebseiten	43,8
Fachgespräche mit Kolleg:innen	35,2
Fachbücher	33,3
Sonstige Quellen	8,6
*Das Thema gesunde Ernährung ist für mich persönlich (%)*	
Gar nicht wichtig	0,0
Eher nicht wichtig	3,8
Eher wichtig	38,5
Sehr wichtig	57,7
*Das Thema Ernährung ist für meine tägliche Arbeit als Ärzt:in (%)*	
Gar nicht wichtig	0,0
Eher nicht wichtig	6,7
Eher wichtig	45,7
Sehr wichtig	47,6

19,1 % der Teilnehmenden boten in ihrer Praxis eine professionelle Ernährungsberatung und -therapie an, die meist von Diätassistent:innen und nur selten von ernährungsmedizinisch qualifiziertem ärztlichen Personal selbst durchgeführt wurde. In den regulären Sprechstunden sprachen 85,7 % der Ärzt:innen mit ihren Patient:innen über Ernährung und ernährungsbezogene Themen. Bevor sie Medikamente verschrieben oder einen chirurgischen Eingriff planten, berieten sie ihre Patient:innen immer (40,2 %) oder häufig (49,0 %) zu einer Änderung ihres Lebensstils, sofern dies indiziert war (Tab. 3).

**Tab. 3:** Detaillierte Angaben zu Anlass, Durchführung und Dauer der von den teilnehmenden Ärzt:innen durchgeführten Ernährungsberatung (*n* = 105)

*In meiner Praxis wird professionelle EB durchgeführt (%)*	
Ja	19,1
Nein	80,9
*Ich berate meine Patient:innen in der regulären Sprechstunde zu Ernährung (%)*	
Ja	85,7
Nein	14,3
*Ich berate meine Patient:innen zu Lebensstilveränderungen, bevor ich eine medikamentöse/interventionelle Therapie einleite (sofern indiziert; %)*	
Nie	1,0
Selten	9,8
Häufig	49,0
Immer	40,2
*Für EB aufgewendete Zeit pro Patient:in (min; Mittelwert (SD))*	
Insgesamt	11,0 (8,7)
Allgemeinmedizin	13,0 (9,7)
Innere Medizin	9,5 (10,2)
Gynäkologie und Geburtshilfe	9,2 (8,9)
Sonstige	9,5 (5,1)
*Häufigste Anlässe für EB (%)*	
Akuttermin	28,6
Separater Termin	26,7
Routine- oder Verlaufskontrolle	58,1
Termin zum Disease-Management-Programm	47,6
Gesundheits-Check-up	49,5
Andere Vorsorgeuntersuchungen	30,5
Spezielle Schulungen	9,5
Weitere	10,5
*Häufigste Themen der EB (%)*	
Spezielle krankheitsbedingte Diäten	35,2
Übermäßige Zufuhr von Energie	73,3
Übermäßige Zufuhr von Fetten	43,8
Übermäßige Zufuhr von Salz	29,5
Übermäßige Zufuhr von Zucker	69,5
Weitere	18,1
*Häufigste Ursachen für EB (%)*	
Adipositas	80,0
Herz-Kreislauf-Erkrankungen	57,1
Hyperlipidämie	47,6
Diabetes mellitus	61,0
Gastrointestinale Erkrankungen	34,3
Osteoporose	21,0
Arthrose	20,0
Rheumatoide Arthritis	14,3
Gicht	41,9
Kinderwunsch und Schwangerschaft	21,0
Polyzystisches Ovarialsyndrom	19,1
Schwangerschaftsdiabetes	26,7
Nahrungsmittelunverträglichkeiten und Allergien	38,1
Psychische Erkrankungen	15,2
Krebserkrankungen	15,2
Weitere	7,6

*EB* Ernährungsberatung

Die Ärzt:innen schätzten, dass durchschnittlich 39,5 % ihrer Patient:innen eine Ernährungsberatung benötigten, mit einer großen Spannweite von 5–100 %. Allgemeinmediziner:innen schätzten den Anteil auf durchschnittlich 41,7 %, Internist:innen auf 37,7 %, Gynäkolog:innen auf 36,7 % und Sonstige auf 42,5 %.

**Abb. 1:**
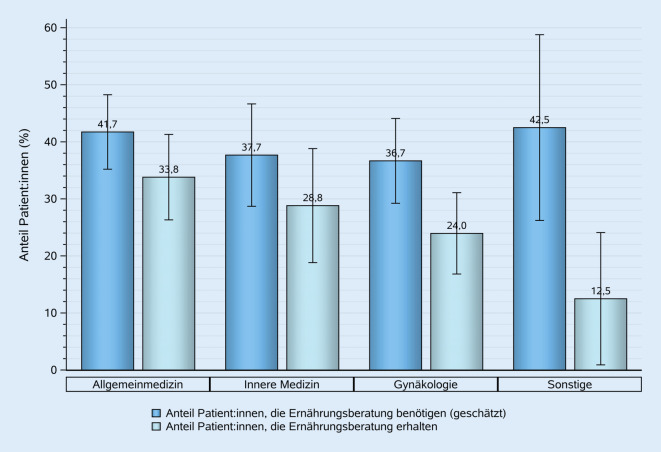
Geschätzter Anteil der Patient:innen, die Ernährungsberatung benötigen, vs. Anteil der Patient:innen, die Ernährungsberatung erhalten, gruppiert nach den Fachgebieten der Ärzt:innen. (Quelle: eigene Abbildung)

Während 39,5 % der Patient:innen den Schätzungen zufolge eine Ernährungsberatung benötigten, wurden im Durchschnitt nur 28,1 % von ihnen auch tatsächlich beraten. Allgemeinmediziner:innen sprachen mit 33,8 % ihrer Patient:innen über Ernährung, Internist:innen lagen mit 28,8 % leicht über dem Durchschnitt. In der Gynäkologie (24,0 %) und den sonstigen Fachgruppen (12,5 %) wurde bei einem deutlich geringeren Anteil der Patient:innen eine Ernährungsberatung durchgeführt (Abb. 1).

Ärzt:innen, die in Gemeinschaftspraxen arbeiteten, berieten einen signifikant größeren Teil ihrer Patient:innen zu Ernährung als Ärzt:innen in Einzelpraxen (*p* = 0,046). Allerdings war der Zeitaufwand für die Ernährungsberatung pro Patient:in bei Ärzt:innen in Einzelpraxen signifikant höher (*p* = 0,009).

Die Ernährungsberatung durch Ärzt:innen dauerte im Durchschnitt 11,0 ± 8,7 min. In der Allgemeinmedizin wurde im Vergleich zu den anderen medizinischen Fachrichtungen deutlich mehr Zeit pro Patient:in dafür aufgewendet (Tab. 3). Die Ernährungsberatung fand zu zahlreichen Anlässen statt, am häufigsten bei Routineuntersuchungen (58,1 %), Gesundheits-Check-ups (49,5 %) und im Rahmen von Disease-Management-Programmen (47,6 %). Die Gespräche konzentrierten sich insbesondere auf die übermäßige Aufnahme von Energie (73,3 %) und Zucker (69,5 %). Spezielle Diäten bei bestimmten Krankheiten und der Verzehr von ungesunden Mengen an Fett und Salz waren ebenfalls ein häufiger Bestandteil. Die häufigsten Krankheiten, die Anlass zu einer Ernährungsberatung gaben, waren Übergewicht und Adipositas (80,0 %) und die damit verbundenen Erkrankungen, wie Diabetes mellitus (61,0 %), Herz-Kreislauf-Erkrankungen (57,1 %) und Hyperlipidämie (47,6 %).

**Abb. 2:**
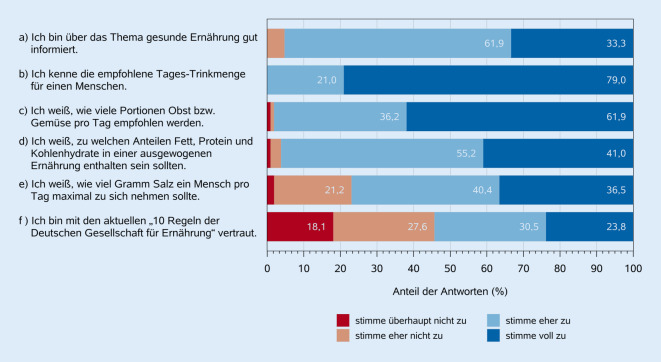
Selbst eingeschätztes Wissen der Ärzt:innen über Grundlagen der Ernährungsmedizin. (Quelle: eigene Abbildung)

Die meisten Teilnehmer:innen hielten eine gesunde Ernährung für sehr (57,7 %) oder eher (38,5 %) wichtig für ihr privates Leben (Tab. 2). Frauen zeigten ein signifikant höheres persönliches Interesse an gesunder Ernährung als Männer (*p* = 0,003). Es bestand große Zustimmung zu den Aussagen, dass Ernährung ein wichtiger Bestandteil der täglichen Arbeit als Ärzt:in ist und dass die eigene Ernährung der Ärzt:innen die Ergebnisse ihrer Ernährungsberatungen beeinflusst. Die Mehrheit der Ärzt:innen fühlte sich über gesunde Ernährung gut informiert. Einzelheiten zum selbst eingeschätzten Wissen der Teilnehmenden siehe Abb. 2.

Es wurde eine mäßige Korrelation zwischen der für die Ernährungsfortbildung aufgewendeten Zeit und dem selbst eingeschätzten Wissen festgestellt (Spearman-Korrelation 0,41; *p* < 0,001). Das selbst eingeschätzte Wissen wurde zu Beginn und am Ende des Fragebogens abgefragt. Während 33,3 % der Aussage: „Ich bin über das Thema gesunde Ernährung gut informiert“, zu Beginn voll zustimmten, stimmten nur 14,3 % Prozent einer ähnlichen Frage („Mein Wissensstand zum Thema Ernährung ist ausreichend hoch“) am Ende voll zu.

59,2 % der Teilnehmenden wendeten weniger als 10 % ihrer jährlichen Fortbildungszeit für ernährungsbezogene Themen auf. Die wichtigsten Informationsquellen zum Thema Ernährung waren (in absteigender Reihenfolge) Fachzeitschriften (70,5 %), Fortbildungen/Tagungen/Kongresse (55,2 %), Fachwebseiten (43,8 %), offizielle Leitlinien (41,9 %), Fachgespräche mit Kolleg:innen (35,2 %) und Fachbücher (33,3 %; Tab. 2).

**Abb. 3:**
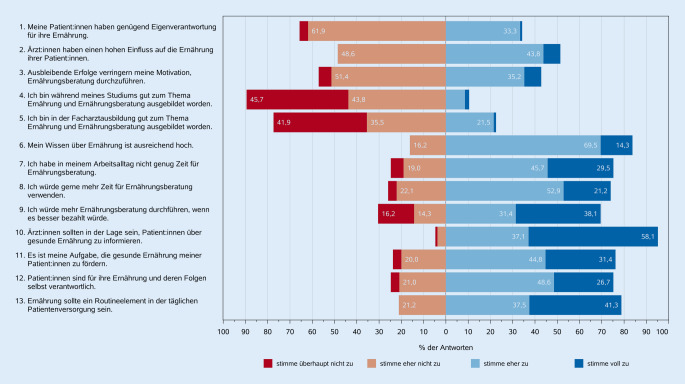
Meinungen der teilnehmenden Ärzt:innen zu Aspekten der medizinischen Ernährungsberatung. (Quelle: eigene Abbildung)

Fast alle Teilnehmenden stimmten zu, dass Ärzt:innen in der Lage sein sollten, ihre Patient:innen über gesunde Ernährung zu informieren. 76,2 % waren der Meinung, dass es in ihrer Verantwortung liegt, gesunde Ernährung bei den Patient:innen aktiv zu fördern. Die meisten Ärzt:innen betonten, dass Patient:innen selbst für ihre Ernährung und deren Folgen verantwortlich sind, waren aber dennoch der Meinung, dass die Ernährung ein Routineelement in der täglichen Patientenbetreuung sein sollte (Abb. 3).

Zwei Drittel aller Teilnehmenden gaben an, dass ihre Patient:innen nicht genügend Eigenverantwortung für die eigene Ernährung aufwiesen. Die Hälfte der Ärzt:innen war der Meinung, einen starken Einfluss auf das Ernährungsverhalten ihrer Patient:innen zu haben. Etwas über 40 % fühlten sich in ihren Beratungsbemühungen entmutigt, weil ihre Patient:innen die vereinbarten Ernährungsziele nicht erreichten (Abb. 3).

Eine große Mehrheit der Ärzt:innen empfand ihre Ausbildung in Bezug auf Ernährung und Beratung im Medizinstudium und in der Facharztausbildung als schlecht und unzureichend. Im Hinblick auf die Facharztausbildung sind Unterschiede zwischen den medizinischen Fachrichtungen zu beobachten: Allgemeinmediziner:innen und Internist:innen bewerteten ihre Ausbildung zu ernährungsmedizinischen Themen und Patient:innenberatung deutlich besser als die anderen Fachrichtungen (*p* = 0,009). 75 % der Ärzt:innen gaben an, in ihrem Arbeitsalltag nicht genügend Zeit für eine angemessene Ernährungsberatung zu haben. Ebenso gaben 75 % der Teilnehmenden an, dass sie gerne mehr Zeit für diese Gespräche hätten, und 70 % würden sie vermehrt durchführen, wenn sie für diese Leistung besser bezahlt würden (Abb. 3).

Fast alle Teilnehmenden waren sich einig, dass eine höhere finanzielle Entlohnung, mehr Zeit für Beratung und eine bessere ernährungsmedizinische Ausbildung während des Medizinstudiums notwendig sind, um Patient:innengespräche über Ernährung in ärztlichen Praxen zu fördern. Darüber hinaus wurden politische Veränderungen hin zu gesünderen Lebensmitteln und Maßnahmen für ein besseres Ernährungsverhalten in der Bevölkerung von über 90 % der Teilnehmenden befürwortet.

## Diskussion

Ziel dieser Studie war es, den Stand der professionellen und nicht-professionellen Ernährungsberatung in ärztlichen Praxen zu ermitteln. Dabei wurde auch das Interesse der Ärzt:innen am Thema Ernährung, ihr Wissen sowie bestehende Hürden und Möglichkeiten zur Verbesserung der Versorgung in ärztlichen Praxen in Deutschland untersucht. Die Ergebnisse zeigten, dass Ärzt:innen sich der Bedeutung von Ernährung und Ernährungsberatung für die Gesundheit ihrer Patient:innen bewusst sind, aber den von ihnen selbst geschätzten Bedarf an Patient:innengesprächen über Ernährung nicht decken. Die Gründe hierfür sind in erster Linie Zeitmangel, fehlende finanzielle Vergütung, unzureichende Ernährungsausbildung während des Studiums und der Facharztweiterbildung und die mangelnde Motivation und Eigenverantwortung der Patient:innen.

Bereits vor 30 Jahren war bekannt, dass Faktoren wie Zeitmangel oder mangelnde Motivation der Patient:innen Gründe für eine unzureichende Beratung von Patient:innen in Ernährungsfragen sein können [25, 26]. Wie Visser et al (2008) zeigten, hatte sich das Bewusstsein der Ärzt:innen für ernährungsbedingte Krankheiten und ihr Verantwortungsgefühl für die Gesundheitserziehung ihrer Patient:innen seit 1992 nicht verändert. Die Ärzt:innen gaben jedoch an, weniger Ernährungsaufklärung als früher durchzuführen, und fühlten sich weniger in der Lage, übergewichtige Patient:innen angemessen zu betreuen. Ähnliche Probleme bestehen unserer und internationalen Studien zufolge auch heute noch [13, 27, 28]. Zeitmangel und mangelnde Vergütung „sprechender Medizin“ stellen dabei grundsätzliche Probleme in der vertragsärztlichen Versorgung in Deutschland dar [29].

Die professionelle Ernährungsberatung und -therapie hat sich inzwischen unter anderem durch zunehmende Standardisierung und Verbesserung der Maßnahmen sowie eine Professionalisierung der Fachkräfteausbildung enorm weiterentwickelt [10]. Durch hohes Fachwissen, höhere zeitliche Kapazitäten und weitere Faktoren haben Ernährungsfachkräfte die Möglichkeit einer umfassenderen Versorgung, wie sie Ärzt:innen im Rahmen ihrer Sprechstunde oft nicht leisten können. Die Zusammenarbeit von Ernährungsfachkräften und Ärzt:innen ist daher für eine bestmögliche Versorgung von Patient:innen mit ernährungsassoziierten Problemen von hoher Relevanz [10, 11].

Die Ausbildung zu Ernährungsthemen sowohl im Studium als auch in der Facharztausbildung wird von den meisten Ärzt:innen als unzureichend bewertet. Interessanterweise bewerteten die meisten Teilnehmenden ihr eigenes Ernährungswissen als gut oder sehr gut, trotz der von ihnen als unzulänglich empfundenen Ausbildung. Es ist davon auszugehen, dass sich viele der teilnehmenden Ärzt:innen ihr Ernährungswissen durch ihr hohes persönliches Interesse an gesunder Ernährung selbstständig angeeignet haben und das erworbene Wissen auch in ihrer Rolle als Ärzt:innen nutzen.

In unserer Studie befassten wir uns überwiegend mit der nichtprofessionellen Ernährungsberatung im Rahmen der alltäglichen Sprechstunde niedergelassener Ärzt:innen. Die meisten internationalen Studien bezogen sich auf die Fachrichtungen Allgemeinmedizin [14, 18, 21, 23] und Innere Medizin [16, 20]. In unsere Studie bezogen wir auch Ärzt:innen der Fachrichtungen Orthopädie, Arbeitsmedizin und Gynäkologie ein, um die Einstellungen, Vorgehensweisen und Kenntnisse eines breiteren ärztlichen Spektrums darzustellen.

Da durch die postalische Rücksendung der Fragebögen Anonymität gewährleistet war, können wir davon ausgehen, dass die Antworten unverfälscht und wahrheitsgemäß waren. Dennoch beruht die Studie auf Selbstauskünften, die anfällig für Verzerrungen sein können. Dies zeigt sich bei den Fragen zum selbst eingeschätzten Ernährungswissen der Teilnehmenden, das möglicherweise überschätzt wurde: Mehr als 30 % der Teilnehmenden stimmten zu Beginn des Fragebogens der Aussage, gut über Ernährung informiert zu sein, voll zu. Am Ende des Fragebogens, nachdem weitere Fragen zu Wissen und Ernährungsberatung beantwortet wurden, stimmten weniger als 15 % einer ähnlichen Aussage voll zu.

Ernährungsberatung zielt vor allem auf die Veränderung von individuellem Essverhalten ab. Für langfristige Erfolge und insbesondere im Sinne der Primärprävention wäre ein stärker auf gesunde Ernährung ausgelegtes Lebensumfeld sehr förderlich. Die Weltgesundheitsorganisation (WHO) und Bündnisse wie die Deutsche Allianz gegen nichtübertragbare Krankheiten fordern seit Langem weitgehende verhältnispräventive politische Maßnahmen, die ungesunder Ernährung und deren Folgen entgegenwirken [8, 9]. So führte eine 2018 in Großbritannien eingeführte Steuer auf stark zuckerhaltige Getränke zu einer Reduktion des Zuckergehaltes um durchschnittlich 43,7 % [30] und erste Erfolge bei der Reduktion von Übergewicht ließen sich darauf zurückführen [31]. Eine bundesweit verpflichtende Anpassung von Kantinenessen an die Vorgaben der Deutschen Gesellschaft für Ernährung würde eine gesunde Ernährung fördern und ließe sich häufig mit nur marginaler Kostensteigerung realisieren [32]. Um ungesunder Ernährung bereits im Kindesalter vorzubeugen, wären ein Verbot von Kindermarketing für ungesunde Lebensmittel und eine verstärkte Schulbildung zu gesunder Ernährung sinnvolle Maßnahmen [8].

Derartige politische Maßnahmen wünschten sich auch über 90 % der teilnehmenden Ärzt:innen unserer Studie. Durch die Kombination aus politischen und gesellschaftlichen Maßnahmen für die Gesamtbevölkerung und Ernährungsberatung für Patient:innen mit entsprechendem Bedarf ließen sich Essgewohnheiten nachhaltig gesünder gestalten und Übergewicht und viele nichtübertragbare Krankheiten könnten effektiver behandelt und vermieden werden.

### Limitationen

Da diese Studie in einer einzigen westdeutschen Stadt durchgeführt wurde, sind die Ergebnisse nur eingeschränkt repräsentativ. Im Hinblick auf die Rücklaufquote von 29,2 % kann ein Selektionsbias nicht ausgeschlossen werden. Es ist möglich, dass die Teilnehmenden eine Gruppe darstellen, die ein überdurchschnittlich hohes Interesse an Ernährung hat, was zu leicht verzerrten Ergebnissen führen kann. Unsere Studie differenziert nicht zwischen Ernährungsgesprächen bei privat- und gesetzlich versicherten Patient:innen. Bezüglich des Zeitaufwandes und der Vergütung könnten hier jedoch Unterschiede bestehen und die Datenlage verzerren.

## Fazit

Die vorliegende Studie zeigt, dass ambulante Ärzt:innen sich ihrer Rolle als oftmals erste Anlaufstelle für Menschen mit ernährungsassoziierten Gesundheitsproblemen bewusst sind. Sie wissen, wie wichtig Patient:innengespräche über Ernährung für die gesundheitliche Versorgung sind, und würden diese unter verbesserten Bedingungen auch verstärkt durchführen. Die Studie soll eine Informationsgrundlage für die Ausweitung der Forschung zur Beratung über Ernährung in ärztlichen Praxen in Deutschland liefern. Für eine erhöhte Aussagekraft könnten zukünftige Studien größere Stichproben aus städtischen und ländlichen Gebieten in ganz Deutschland einbeziehen und über offizielle Kanäle, beispielsweise der kassenärztlichen Vereinigungen, verbreitet werden. Für ein umfassendes Bild könnten zukünftige Studien auch die Perspektiven von Patient:innen und professionellen Ernährungsfachkräften einbeziehen und die Schnittstellen zwischen dem ärztlichen Gespräch über Ernährung und professioneller Ernährungsberatung und -therapie stärker in den Fokus nehmen.

Rund 90 % der Teilnehmenden dieser Studie würden Veränderungen wie eine bessere finanzielle Vergütung der Ernährungsberatung durch die Krankenkassen, mehr Zeit für ihre Patient:innen zur ausführlichen Besprechung der Ernährungsprobleme und die Aufnahme der Ernährungsmedizin in die Lehrpläne von Medizinstudierenden begrüßen. Die Teilnehmenden sprachen sich zusätzlich für mehr politische Initiativen zur Förderung gesunder Ernährung aus, um ein gesundes Essverhalten ihrer Patient:innen nachhaltig zu fördern und zur Prävention von Krankheiten beizutragen.
